# The ataxia-linked E1081Q mutation affects the sub-plasma membrane Ca^2+^-microdomains by tuning PMCA3 activity

**DOI:** 10.1038/s41419-022-05300-y

**Published:** 2022-10-07

**Authors:** Francesca Vallese, Lorenzo Maso, Flavia Giamogante, Elena Poggio, Lucia Barazzuol, Andrea Salmaso, Raffaele Lopreiato, Laura Cendron, Lorella Navazio, Ginevra Zanni, Yvonne Weber, Tatjana Kovacevic-Preradovic, Boris Keren, Alessandra Torraco, Rosalba Carrozzo, Francesco Peretto, Caterina Peggion, Stefania Ferro, Oriano Marin, Giuseppe Zanotti, Tito Calì, Marisa Brini, Ernesto Carafoli

**Affiliations:** 1 Department of Biomedical Sciences, University of Padova, Padova, Italy; 2 Department of Biology, University of Padova, Padova, Italy; 3 Department of Neurosciences, Bambino Gesù Children’s Hospital, IRCCS, Rome, Italy; 4 Department of Neurology and Epileptology, Hertie Institute for Clinical Brain Research, University of Tübingen, Tübingen, Germany; 5 Epilepsiezentrum Kleinwachau gemeinnützige GmbH, Radeberg, Germany; 6 Département de Génétique Hôpital Pitié-Salpêtrière, Paris, France; 7 Centro Studi per la Neurodegenerazione (CESNE), University of Padova, Padova, Italy; 8 Padova Neuroscience Center (PNC), University of Padova, Padova, Italy; 9 Venetian Institute of Molecular Medicine, Padova, Italy; 10 Present Address: Department of Anesthesiology, Columbia University Irving Medical Center, New York, USA

**Keywords:** Calcium channels, Neurochemistry

## Abstract

Calcium concentration must be finely tuned in all eukaryotic cells to ensure the correct performance of its signalling function. Neuronal activity is exquisitely dependent on the control of Ca^2+^ homeostasis: its alterations ultimately play a pivotal role in the origin and progression of many neurodegenerative processes. A complex toolkit of Ca^2+^ pumps and exchangers maintains the fluctuation of cytosolic Ca^2+^ concentration within the appropriate threshold. Two ubiquitous (isoforms 1 and 4) and two neuronally enriched (isoforms 2 and 3) of the plasma membrane Ca^2+^ATPase (PMCA pump) selectively regulate cytosolic Ca^2+^ transients by shaping the sub-plasma membrane (PM) microdomains. In humans, genetic mutations in *ATP2B1, ATP2B2* and *ATP2B3* gene have been linked with hearing loss, cerebellar ataxia and global neurodevelopmental delay: all of them were found to impair pump activity. Here we report three additional mutations in *ATP2B3* gene corresponding to E1081Q, R1133Q and R696H amino acids substitution, respectively. Among them, the novel missense mutation (E1081Q) immediately upstream the C-terminal calmodulin-binding domain (CaM-BD) of the PMCA3 protein was present in two patients originating from two distinct families. Our biochemical and molecular studies on PMCA3 E1081Q mutant have revealed a splicing variant-dependent effect of the mutation in shaping the sub-PM [Ca^2+^]. The E1081Q substitution in the full-length *b* variant abolished the capacity of the pump to reduce [Ca^2+^] in the sub-PM microdomain (in line with the previously described ataxia-related PMCA mutations negatively affecting Ca^2+^ pumping activity), while, surprisingly, its introduction in the truncated *a* variant selectively increased Ca^2+^ extrusion activity in the sub-PM Ca^2+^ microdomains. These results highlight the importance to set a precise threshold of [Ca^2+^] by fine-tuning the sub-PM microdomains and the different contribution of the PMCA splice variants in this regulation.

## Introduction

The P-type Ca^2+^ ATPases of the plasma membrane (PMCA) [1], along with the Na^+^/Ca^2+^exchanger and the SERCA pump, are in place to maintain basal cytosolic Ca^2+^ levels in the ∼100 nM physiological range by removing Ca^2+^ from the intracellular side [2]. However, the PMCA pumps role in the regulation of the global cytosolic Ca^2+^ homeostasis in most cells is quantitatively minor with respect to that of the two other systems; they rather act as master regulators of Ca^2+^ homeostasis, and thus of Ca^2+^ signalling, in selected sub-PM microdomains [3]. Structurally the PMCA pumps consist of ten transmembrane helices, two intracellular loops and a C-terminal tail containing the calmodulin-binding domain (CaM-BD) which, by interacting with the main body of the pump, inhibits it at resting conditions, i.e., when local Ca^2+^ falls to levels well below 100 nM. When local Ca^2+^ increases, Ca^2+-^saturated CaM that binds to the CaM-BD, removing it from the main body of the pump which becomes active. [4–6]. Four genes (*ATP2B1-4*) encode ubiquitous (PMCA1 and 4) and neuron-enriched (PMCA2 and 3) isoforms, which differ in their activity and compartmentalization at the PM. Alternative splicing in the first cytosolic loop (site A) and within the CaM-BD (site C) greatly increases the number of pump variants [7].

In the nervous system, PMCA3 concentrates in the cerebellum [8, 9], in the choroid plexes and the hippocampus [10]. Genetic defects in *ATP2B1, ATP2B2* and *ATP2B3* genes have been linked to specific neuronal disease phenotypes. The first mutations identified were in the *ATP2B2* gene and have been linked to hereditary deafness [11–19] in mice [16, 17] and in humans [14, 19]. In addition to deafness, PMCA2 mutant mice also have motility and balance problems [11, 13, 20]. Interestingly, a V1143F missense mutation in *ATP2B2* gene product, the PMCA2 pump, has been described in an ataxic patient showing no overt signs of deafness [21]. Defects of PMCA3 associated with X-linked ataxias have been described in rats and humans [22–27]. The first mutation identified in humans has been mapped the CaM-BD (a G1107D substitution) and then additional mutations were found in the catalytic P-domain (R482H and G733R replacements). All the amino acids substitutions identified so far have been found to reduce Ca^2+^-extrusion activity of the pump either by acting on its basal autoinhibition or on the interplay between calmodulin and its CaM-BD. A mutation in the N-terminal domain of the rat PMCA3 has also been described (R35C), however, no effect of the mutation on the Ca^2+^-extrusion function of the pump was found [24]. More recently, a cohort of 12 unrelated individuals with variants in *ATP2B1* and an overlapping phenotype of mild to moderate global development delay has been described, suggesting that PMCA defects in Ca^2+^ extrusion activity play a crucial role in the pathogenicity of a range of neurodevelopmental disorders [28].

In the present study, we describe three different *ATP2B3* novel missense mutations potentially associated with patient’s clinical phenotype compatible with cerebellar ataxia. The three amino acids substitutions are located in the stalk region of the pump upstream the P-domain (R696H), immediately upstream its CaM-BD (E1081Q) and its C-terminal tail (R1133Q), respectively.

Among them, the E1081Q substitution, which is in a highly acidic region immediately upstream of the CaM-BD, strongly affected the ability of the pump to manage cytosolic Ca^2+^ transients generated upon cell stimulation. On the contrary the R696H and the R1133Q replacement failed to cause global changes in the PMCA3 Ca^2+^ extrusion activity.

Unexpectedly, the E1081Q mutation affected the pump function in an isoform-dependent manner: it increases the basal activity of the isoform *a* of the PMCA3, the truncated splicing variant, but decreases that of the full-length *b* variant which contains the entire CaM-BD. To better characterize this aspect, we analysed the following different parameters in cells overexpressing either the truncated splicing variant *a* or the full length splicing variant *b*: i), PMCA E1081Q expression and plasma membrane localization; ii), the return to baseline of the Ca^2+^ transient induced by cell stimulation with an inositol-1,4,5 trisphosphate (InsP_3_) generating agonist in cells overexpressing the mutated pumps; iii), the ability of the mutated pumps to control the Ca^2+^ released from the intracellular stores (ER and Golgi); iv) the effect of mutated pump overexpression on the ER Ca^2+^ content, v) the ability of the mutated pumps to counteract the capacitative Ca^2+^ entry [29] and, vi) their ability to shape sub-PM Ca^2+^-microdomains. Interestingly, the ability of the PMCA3 to specifically modulate sub-plasma membrane Ca^2+^ microdomains was selectively affected in isoform dependent manner, resulting the PMCA3*a* E1081Q more active, and the PMCA3*b* E1081Q variant less, than the WT counterpart. This aspect is completely new, and its relevance in respect with the appearance of neurological phenotype needs to be considered.

## Results

### Clinical description of the patients harbouring *ATP2B3* mutation

**Fig. 1:**
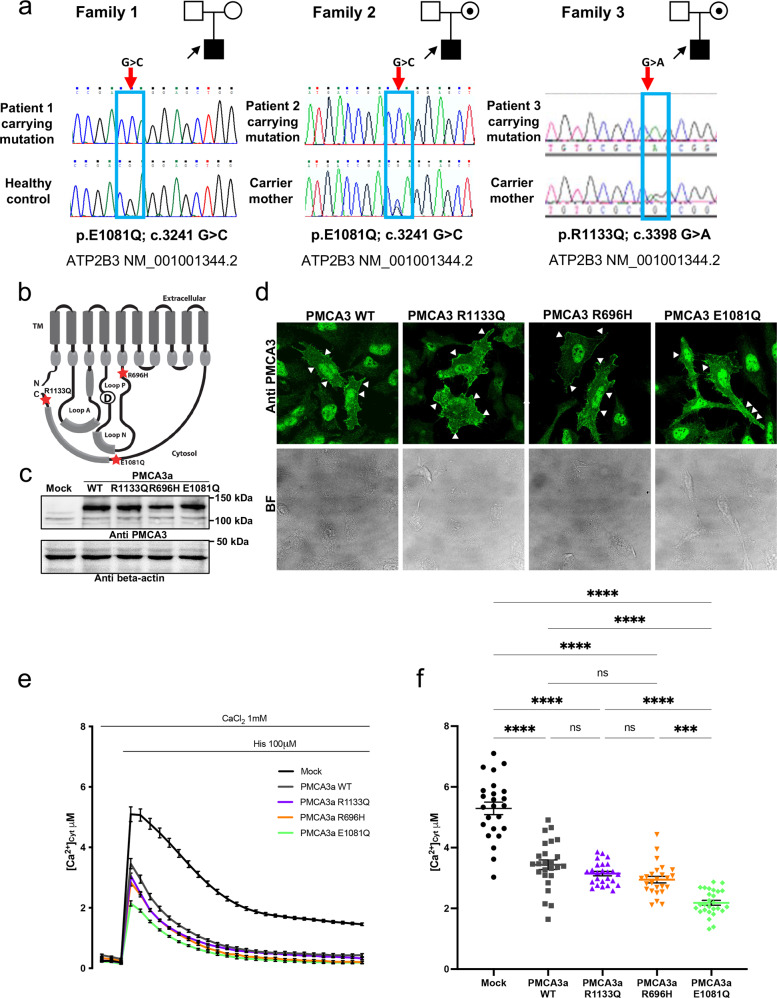
Analysis of the human exogenous pump mutants overexpressed in model HeLa cells and cytosolic Ca^2+^ measurements. **a** Pedigree of the families with three affected males suggesting X-linked inheritance in family 2 and 3; Sanger sequencing chromatograms showing PMCA3 mutations in the patients and control subjects. **b** Schematic representation of PMCA topology with selected point mutations position marked with a red star. **c** Western blotting and **d** immunocytochemistry, analysis showing the expression level and cellular localization of the wild type and mutated PMCA3. The PMCA3 was revealed by the rabbit polyclonal anti-PMCA3 antibody. **e** Cytosolic Ca^2+^ transients recorded following 100 μM histamine stimulation of HeLa cells overexpressing the cytAEQ either alone or along with the wt or the mutants PMCA3a pump. **f** Average peak [Ca^2+^] values measured upon stimulation (bars represent mean μM ± SEM). ***, *p* < 0,001; ****, *p* < 0.0001: ns, not significant. The numbers on the dots indicate the number of independent measurements out of three independent transfections.

Herein, we describe three novel mutations in *ATP2B3* found in four unrelated patients presented with clinical features varying widely and ranging from the neurodevelopmental delay with early–onset seizures to acute and severe neurological disease with psychomotor regression in the first years of life. Patient 1 and 2 both carried p.E1081Q; c.3241 G > C mutation in the *ATP2B3* gene NM_001001344.2 (Fig. 1a), that in the case of patient 2 was inherited by the healthy mother.

The patient from family 1 showed leukodystrophy with clinical signs of acute psychomotor regression, the outcome was respiratory failure, swallowing disturbances and epileptic seizures, The patient carried biallelic compound heterozygous variants in the *IBA57* gene (NM_001010867.3); c.87ins_GCCCAAGGTGC; p.R30Afs*46 and c.313 C > T; p.R105W,both located in exon 1 and inherited from heterozygous parents. Brain MRI previously reported diffuse leukoencephalopathy extended to the corpus callosum and posterior arm of the internal capsulae, cavitations of the white matter marked posteriorly, signal abnormalities in the posterior column of the cervical spinal cord [30]. The patient from family 2, carrier of a *de novo* variant in the *SCN1A* gene, NM_001165963: c.3971 T > C, p.L1324P, presented neurodevelopmental delay and early-onset seizures. Brain MRI showed hypoplasia of the inferior portion of the cerebellar vermis with enlargement of the cisterna magna (Figure S1).

Regarding the patient from family 3, harbouring a c.3398 G > A:p.R1133Q variant in the *ATP2B3* gene (NM_001001344.2) inherited from his healthy mother (Fig. 1a) and a rare pathogenic variant in *SLC2A1* NM_006516.4:c.805 C > T; p.R269C. He presented neurodevelopmental delay, tonic seizures, with onset at 3 years of age, complex partial seizures, GTCS (generalized tonic-clonic seizures) and moderate intellectual disability (ID) with normal brain MRI (Figure S1).

As for patient 4, harboring a c.2087 G > A; p.R696H variant in the *ATP2B3* gene (NM_001001344.2), no clinical data are available and further analysis revealed that the variant represents a rare polymorphism (https://www.ncbi.nlm.nih.gov/snp/rs782459941).

### Expression, subcellular localization of the wt, the R696H, the E1081Q and the R1133Q mutant PMCA3 pumps and ability to clear the InsP_3_-induced Ca^2+^ transient

Considering our records of disease-related *ATP2B3/ATP2B2* mutations [21, 24–27], we were interested in analysing the effect of these three novel mutations on pump activity, including the R696H polymorphism as an internal control. Figure 1b shows a cartoon of PMCA membrane topology and the position of the three amino acids substitutions. The first mutation is located in the P-domain (R696H), the second immediately upstream the CaM-BD (E1081Q) and the third one in the C-terminal tail (R1133Q) respectively (Fig. 1b). To explore their effect on pump’s ability to pump Ca^2+^, the wt human splice variant (*a*) and the mutant pumps were transfected in HeLa cells and their expression levels were determined by Western blot analysis. As shown in Fig. 1c, the overexpressed pumps migrated at the expected molecular weight of about 130 kDa and no difference was detected in their expression level compared to the wt counterpart (Figure S2). The distribution of the pump was also not affected by the mutations as all the overexpressed mutants showed specific enrichment at the plasma membrane as shown in Fig. 1d and pointed by the arrows on transfected cells. The nuclear fluorescence is evidently a spurious signal, since it is also visible in untransfected cells, where instead no fluorescence on the plasma membrane was present.

To explore the effect of the mutations on the Ca^2+^ export activity, HeLa cells were transfected with the wt or the 3 mutant R696H, E1081Q and R1133Q pumps along with a vector encoding the cytosolic photoprotein aequorin (cytAEQ) and were stimulated with the InsP_3_-linked agonist histamine (100 μM) to induce Ca^2+^ release from the ER. As shown by the Ca^2+^ transients of Fig. 1e and the peaks quantified in Fig. 1f, cells overexpressing the wt PMCA3a pump cleared the histamine-induced Ca^2+^ transient more efficiently than control cells (peak values μM ± SEM: 5,29 ± 0,20, n = 24 for control cells; 3,43 ± 0,15, n = 25 for wt PMCA3a). Cells overexpressing the R1133Q and the R696H mutant pumps were as efficient as the cells expressing the WT pump in clearing the histamine-induced Ca^2+^ transient, indicating that their Ca^2+^ extrusion activity was not significantly affected by the mutation (peak values μM ± SEM: 3,14 ± 0,07, n = 25 for PMCA3a R1133Q and 2,94 ± 0,10, n = 25 for R696H PMCA3a). The amplitude of the Ca^2+^ peak generated by histamine cell stimulation in cells overexpressing the PMCA3a E1081Q mutant pump was instead significantly lower compared to that of the cells overexpressing the wt pump (peak values μM ± SEM: 3,43 ± 0,15, n = 25 for wt PMCA3a and 2,18 ± 0,07, n = 27 for E1081Q mutant pump). Surprisingly, the Ca^2+^ ejection activity of the pump was enhanced by the mutation. We therefore concentrated on the characterisation of the effects of this mutation to explore possible mechanism(s) underpinning the increased Ca^2+^ ejection ability of the E1081Q mutation.

### Effect of the E1081Q mutation on the Ca^2+^ ejection of the PMCA3 pump from the ER, from the extracellular ambient, and on the basal ER Ca^2+^ level

**Fig. 2:**
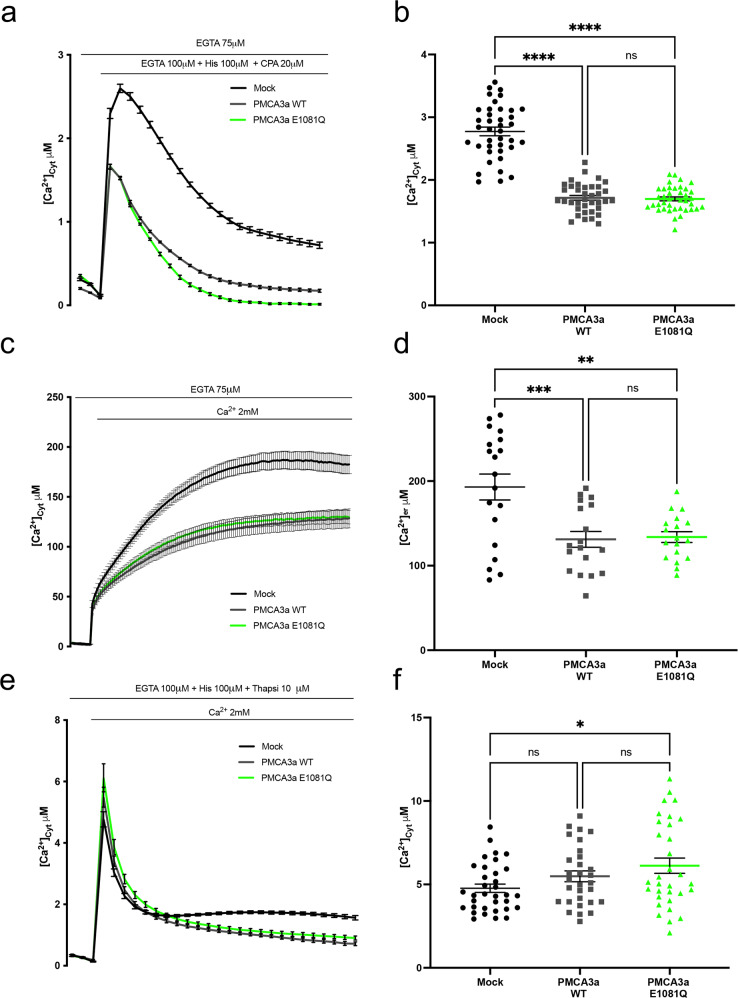
Comparison between human PMCA3a wt and mutant E1081Q activity on cytosolic and ER Ca^2+^ handling. **a**, **b** Kinetic of ER release of the full-length wt and E1081Q mutant pump. HeLa cells were co-transfected with cytAEQ and the PMCA3a constructs stimulated with histamine 100 μM and CPA (20 μM) and in the absence of extracellular Ca^2+^ (100 μM EGTA). **c**, **d** HeLa cells were co-transfected with erAEQ (targeted to the ER lumen) and the PMCA3a constructs. ER refilling upon re-addition of 1 mM CaCl_2_ to Ca^2+^-depleted cells in HeLa cells either mock transfected or overexpressing PMCA3a wt or E1081Q. **e**, **f** Effect full length PMCA3a and mutant E1081Q pump on the influx of Ca^2+^ from the extracellular medium. HeLa cells were co-transfected with cytAEQ and the PMCA3a constructs. All the traces refer to representative experiments selected from at least three independent experiments. Quantifications for panels **a**–**c** are shown respectively in panels **b**–**f**. Bars represent means ± SEM obtained by averaging the values obtained in at least 30 independent measurements from three independent transfections for each condition. *, p < 0.05: **, *p* < 0.01: ***, *p* < 0.001; ****, *p* < 0.0001: ns not significant.

The Ca^2+^ transients generated by histamine stimulation in 1 mM extracellular Ca^2+^ shown above are the sum of the Ca^2+^ released from the ER and Golgi, and the Ca^2+^ influx through the store-operated Ca^2+^ entry (SOCE). We decided to analyse the two components separately to better evaluate the effect of the mutation on the Ca^2+^ extrusion ability of the PMCA pump (Fig. 2): Ca^2+^ release from the intracellular stores were elicited by histamine in the absence of extracellular Ca^2+^ (in Krebs Ringer Buffer, KRB supplemented with 100 μM EGTA and 20 μM CPA) (Fig. 2a, b), and Ca^2+^ influx was monitored in cells treated with histamine in the presence of 100 μM EGTA and 10 μM thapsigargin followed by Ca^2+^ re-addition (Fig. 2e, f). As shown and quantified in Fig. 2a, b, the overexpression of either the wt or the E1081Q mutant pump reduced the amplitude of the Ca^2+^ peak, but the cells expressing the E1081Q mutant pump showed no difference in the peak values compared to those expressing the wt PMCA3a, indicating that the mutation did not compromise the Ca^2+^ extrusion ability of the pump (peaks values ± SEM: 2,77 ± 0,06, *n* = 39 for control cells; 1,71 ± 0,03, *n* = 37 for the wt PMCA3a pump; 1,69 ± 0,03, *n* = 39 for the E1081Q mutant PMCA3a). However, it is evident that Ca^2+^ transient after the peak sets to a lower basal level in PMCA3a E1081Q than in cells overexpressing PMCA3a wt pump. Since we had previously shown that the overexpression of the PMCA pumps could affect the basal ER Ca^2+^ content [26, 29, 31], we assessed whether the expression of the E1081Q mutant PMCA3a impacts on ER Ca^2+^ levels. To this end the ER targeted low affinity aequorin (erAEQ) was expressed in HeLa cells either alone or together with the wt or the E1081Q mutated pump. As shown and quantified in Fig. 2c, d, in agreement with previous reports [26, 31], the maximum [Ca^2+^]_ER_ reached in control cells was higher than in those observed in cell overexpressing the PMCA3a pump, both wt and E1081Q (plateau values ± SEM: 193,0 ± 15,37 μM, *n* = 19 for control cells; 131,1 ± 9,28 μM, *n* = 18 for the wt PMCA3a pump; 133,8 ± 6,37 μM, *n* = 18 for the E1081Q PMCA3a pump). The finding that the ER Ca^2+^ content was not changed in cells overexpressing the wt pump compared to those overexpressing the mutant PMCA3a suggests that the mutation did not modify the ability of the pump to influence the ER Ca^2+^ filling or its basal level.

To gain further insights into the role of the E1081Q mutation on the pump activity and having excluded a role of the ER Ca^2+^ handling, we applied a different protocol. We pre-depleted the intracellular Ca^2+^ deposits by treating cells for 5 minutes with thapsigargin and histamine in a medium containing 1 mM EGTA. Then, upon the addition of a solution containing 2 mM CaCl_2_ which induces fast Ca^2+^ entry from the extracellular ambient, we exclusively monitored the effect of PMCA on Ca^2+^ influx. As shown and quantified in Fig. 2e, f the peak generated under these conditions was higher than that obtained upon histamine stimulation in the presence of extracellular Ca^2+^ and in the absence of SERCA pumps inhibitors (Fig. 1e, f) and was marginally affected by the overexpression of the mutated pump or the wt pump (peak values ± SEM: 4,76 ± 0,24 μM, *n* = 34 for control cells; 5,48 ± 0,32 μM, *n* = 30 for the wt PMCA3a pump; 6,12 ± 0,45 μM, *n* = 32 for the E1081Q PMCA3a pump). This was not surprising as under these conditions the massive opening of Ca^2+^ influx channels could easily overwhelm the PMCA activity. When the Ca^2+^ peaks declined, a plateau was reached (Fig. 2e) at about 1.5 µM Ca ^2+^ in control cells: at this cytosolic Ca^2+^ threshold the Ca^2+^ extrusion activity of the pump became appreciable in the overexpressing cells. No difference was observed in cells overexpressing the wt or the mutated pump, suggesting that under those conditions the E1081Q mutation did not affect the activity of the PMCA3a pump. Since the above-mentioned experiments were performed by using the C-terminally truncated splice variant (*a*), to further explore the role of the E1081Q mutation on the pump activity, the same mutation was introduced in the full-length (*b*) variant of the PMCA3 and its effects on Ca^2+^ extrusion activity of the pump further evaluated.

### Effect of the E1081Q mutation on the Ca^2+^ ejection ability of the full-length PMCA3*b* pump

The experiments above have indicated that the introduction of a E1081Q substitution activates the Ca^2+^ pumping ability of the PMCA3*a* variant to oppose the histamine-induced Ca^2+^ transients compared to its wt counterpart. Therefore, we decided to check whether the E1081Q mutation could impact on the full-length *b* variant of the PMCA3 differently. To this aim we have introduced the point mutation in the rat full-length *b* variant of the PMCA3 and, in analogy to what we did for the human C-terminally truncated *a* variant, we checked its effect on the expression, subcellular localization and Ca^2+^ handling.

**Fig. 3:**
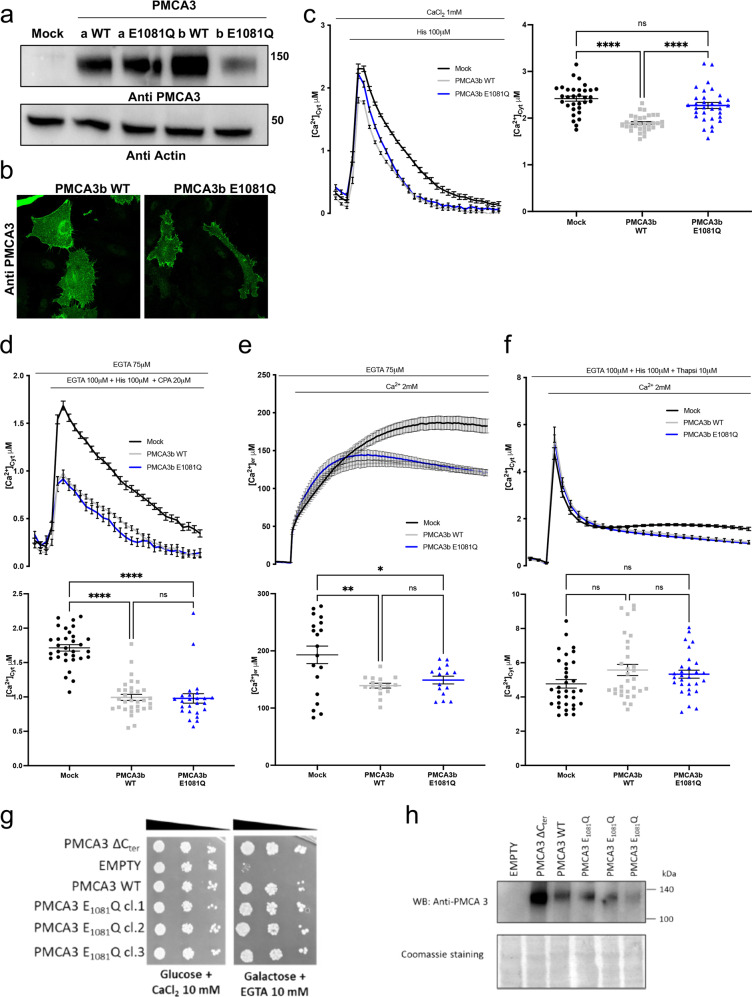
Effect of the E1081Q mutation on PMCA3b pump expression levels, distribution and cytosolic and ER Ca^2+^ handling. **a** Western blotting to compare the expression level of wt and mutant E1081Q PMCA3a and PMCA3b pumps; **b** immunocytochemistry analysis showing the cellular localization of the wt and mutated PMCA3b. The PMCA3b was revealed by the rabbit polyclonal anti-PMCA3 antibody. **c** Cytosolic Ca^2+^ transients recorded following 100 μM histamine stimulation of HeLa cells overexpressing cytAEQ either alone or along with the wt or the E1081Q PMCA3b pump. **d** Kinetic of ER release of the PMCA3b wt and E1081Q mutant pump. HeLa cells were co-transfected with cytAEQ and the PMCA3b constructs stimulated with histamine 100 μM and CPA (20 μM) and in the absence of extracellular Ca^2+^ (100 μM EGTA). **e** ER refilling upon re-addition of 1 mM CaCl_2_ to Ca^2+^-depleted cells in HeLa cells either mock-transfected or overexpressing PMCA3b wt or E1081Q. HeLa cells were co-transfected with erAEQ. **f** Effect of the wt PMCA3b and mutant E1081Q pump on the influx of Ca^2+^ from the extracellular medium. HeLa cells were co-transfected with cytAEQ, pretreated with 10 μM thapsigargin and 100 μM histamine in the presence of 100 μM EGTA for 3 min and perfused in the presence of KRB/Ca^2+^ 2 mM to stimulate Ca^2+^ entry from the extracellular ambient. All the traces refer to representative experiments selected from at least three independent experiments. Bars represent mean μM ± SEM. *p < 0,05; ***, *p* < 0,001; ****, *p* < 0.0001: ns, not significant. The numbers on the dots indicate the number of independent measurements out of three independent transfections. **g** In vivo complementation assay in K616 yeast cells. Serial dilutions and **h** Western blotting analysis of yeast K616 cells transformed with the indicated vectors. Total protein lysates from yeast cells carrying the indicated pYES2-derived plasmids were probed with the anti-PMCA3 antibody.

The expression levels and the subcellular localization of the wt and E1081Q mutant *a* and *b* variants of the PMCA3 pump were compared by Western blot analysis and immunocytochemistry, respectively. As shown in Fig. 3a (and 1c) the mutation did not affect the expression level of the C-terminally truncated *a* variant, however, to our surprise, the expression level of the full-length *b* variant was strongly affected by the E1081Q mutation (Figure S2). Instead, the subcellular distribution at the plasma membrane was maintained (Fig. 3b). We then evaluated the effect of the mutated *b* variant of the PMCA3 on cytosolic Ca^2+^ transients generated by histamine stimulation in the presence of extracellular Ca^2+^. As shown by the traces and the quantification of Ca^2+^ peaks in Fig. 3c, cells overexpressing the wt PMCA3*b* pump cleared the histamine-induced Ca^2+^ transient more efficiently (peak values ± SEM: 2,41 ± 0,05, *n* = 32 for control cells; 1,89 ± 0,03, *n* = 32 for wt PMCA3*b*), however, cells overexpressing the mutated E1081Q *b* variant substantially reduced their ability to extrude Ca^2+^, as the average Ca^2+^ peak value was equal to that measured in control cells (peak values ± SEM: 2,41 ± 0,05, *n* = 32 for control cells; 2,27 ± 0,06, *n* = 32 for E1081Q PMCA3b). At first glance, on the contrary of what found with the human truncated PMCA3*a* variant, these results might suggest that the mutation completely abolished the pump activity of the full-length *b* variant by affecting its ability to extrude Ca^2+^ in the extracellular medium. However, it must be noticed that the decay of the traces in Fig. 3a is similar for cells expressing the PMCA3*b* wt and the mutant and faster than in the control cells, indicating that also the E1081Q PMCA3*b* mutant retains some Ca^2+^ extrusion activity. It must be also considered that the low expression of the mutated protein might explain this phenotype. On the other hand, when cytosolic Ca^2+^ transients were monitored in cells stimulated with histamine in the absence of extracellular Ca^2+^ (in a medium containing 100 μM EGTA and 20 μM CPA, i.e. also in the presence of a SERCA pump inhibitor), the overexpression of either the wt or the mutated pump reduced the amplitude of the peak to the same extent in respect to control cells, as shown in Fig. 3d, despite their difference in expression level. Indeed, the cells expressing the wt and the E1081Q mutant pump showed no difference in the ability to counteract the Ca^2+^ fluxes deriving from the intracellular stores (peak values ± SEM: 1,71 ± 0,04, *n* = 32 for control cells; 0,99 ± 0,04, *n* = 32 for the wt PMCA3*b* pump; 0,97 ± 0,06, *n* = 32 for the E1081Q mutant PMCA3*b*).

We also assessed the effect of mutant PMCA3*b* expression on the free [Ca^2+^] in the ER lumen. As shown in Fig. 3e and quantified in the panel below, the maximum [Ca^2+^]_ER_ reached in control cells was significantly higher than in those observed in cell overexpressing the PMCA3b pump (plateau values ± SEM: 193,0 ± 15,37 μM, *n* = 19 for control cells; 139,2 ± 4,22 μM, *n* = 16 for the wt PMCA3*b* pump; 149,0 ± 6,81 μM, *n* = 15 for the E1081Q PMCA3*b* pump); however, it was not different in cells overexpressing the wt or the mutant PMCA3*b* pump; suggesting, again, that the mutation does not impinge on the ability of the pump to influence the ER Ca^2+^ filling or its basal level.

The Ca^2+^ transients were also monitored in cells treated with histamine in KRB supplemented with 100 μM EGTA and 10 μM thapsigargin followed by Ca^2+^ re-addition. As shown in Fig. 3f, neither the magnitude of Ca^2+^ influx nor the cytosolic plateau levels were affected by the overexpression of the wt and the mutated pump (Fig. 3f) (values ± SEM: 4,76 ± 0,24 μM, *n* = 34 for control cells; 5,57 ± 0,32 μM, *n* = 31 for the wt PMCA3b pump; 5,33 ± 0,23 μM, *n* = 29 for the E1081Q PMCA3b pump).

The effect of the E1081Q mutation on the basal activity of the PMCA3b pump was also investigated by assessing the effect of ectopic expression of an active PMCA on the lethality of the mutant K616 yeast strain in Ca^2+^-free medium [22, 24, 27]. The K616 strain is a triple mutant yeast, which lacks the main endogenous active Ca^2+^ transport systems, it grows normally in high-Ca^2+^ media but is unable to grow in Ca^2+^-free conditions unless an active Ca^2+^ pump is expressed ectopically: growth rescue in Ca^2+^-free conditions will be indicative of the pump ability to mobilize Ca^2+^. Yeast cells were transformed with the galactose-inducible pYES plasmids encoding either the wt, E1081Q PMCA3b or the constitutively active PMCA3-ΔC_ter_ variant [22]. As shown in Fig. 3g, yeast growth in the permissive medium (CaCl_2_) was identical for yeast cells expressing the wt or mutant PMCA3 protein, in Ca^2+^-depleted medium the E1081Q substitution did not affected cell viability, as indicated by the growth rate of cells expressing the mutant PMCA3 with respect to yeast expressing the wt pump. The expression levels of the mutated pump detected by Western blot analysis, in agreement with the results showed above in mammalian cells, were also affected compared to the amounts of wild-type isoform (Fig. 3g, h). The data from the functional assay in yeast cells thus support the finding that the E1081Q replacement does not affect the basal activity of the PMCA3 pump.

### Effect of the E1081Q mutation of the *a* and *b* PMCA3 variants on the formation of the sub-plasma membrane Ca^2+^ microdomains

**Fig. 4:**
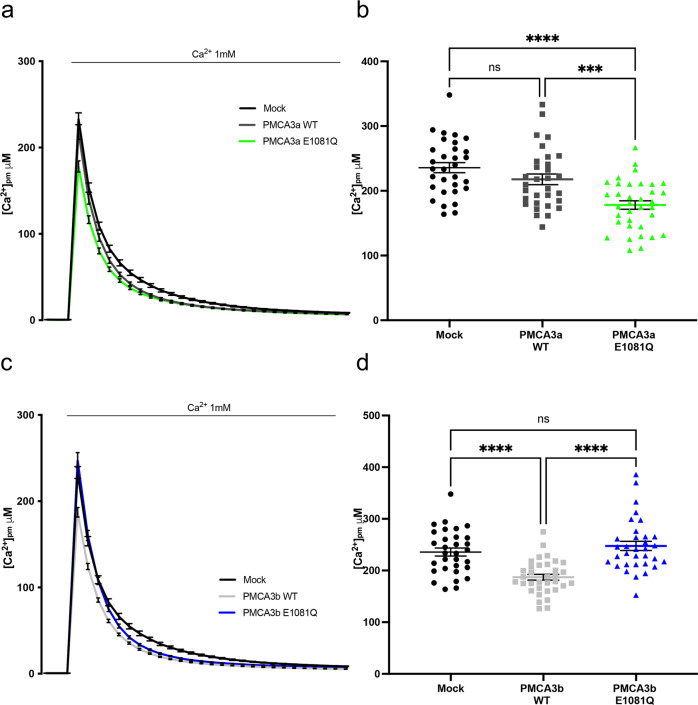
Analysis of the sub-plasma membrane Ca^2+^ microdomains in HeLa overexpressing wt or E1081Q PMCA3*a* or3*b* variants. HeLa cells were co-transfected with pmAEQ (targeted to sub-PM region) and the PMCA3 wt and E1081 mutants constructs for the two splicing variants *a* **a**, **b** and *b* **c**, **d**. Subplasmalemmal region ([Ca^2+^]_pm_) was monitored after a reconstitution with coelenterazine in Ca^2+^-free KRB and and perfused in the presence of KRB/Ca^2+^ 1 mM **a**–**c**. The average peak values are shown in **b**–**d**. Bars represent means ± SEM obtained by averaging the values obtained in at least 30 independent measurements from three independent transfections for each condition. ***, *p* < 0.001; ****, *p* < 0.0001: ns not significant.

As mentioned repeatedly in this contribution, an important role of the PMCA pumps and their splicing variants is the subtle, possibly tissue specific, fine-tuning of Ca^2+^ in selected sub-plasma membrane microdomains. To explore whether the E1081Q mutation could influence the formation of these Ca^2+^ microdomains, HeLa cells were co-transfected with the expression vectors for either the wt or the E1081Q mutated *a* and *b* variants of the PMCA3 pump and the pmAEQ protein, which localized to the plasma membrane after the post-translational addition of a lipid anchor, and the Ca^2+^ concentration in the subplasmalemmal region ([Ca^2+^]_pm_) was monitored. Ca^2+^ influx through plasma membrane channels was induced by perfusing KRB supplemented with 1 mM CaCl_2_. Figure 4a and b shows that, despite the trend towards a reduction, significant differences in [Ca^2+^]_pm_ between control HeLa cells and wt PMCA3a expressing cells could not be appreciated. On the other side, a strong and significant difference in the formation of the Ca^2+^ microdomain in the sub-PM region could be observed upon overexpression of the E1081Q PMCA3*a* pump mutant (peak values ± SEM: 235,7 ± 7,77 μM, *n* = 31 for control cells; 217,8 ± 8,30 μM, *n* = 31 for the wt PMCA3a pump; 178,1 ± 6,51 μM, *n* = 35 for the E1081Q PMCA3a pump), suggesting the possibility that the mutation positively influenced the activity of the truncated *a* but not the full-length *b* PMCA3 splicing variant by selectively affecting the ability to locally control [Ca^2+^]_pm_. Interestingly, a different scenario emerged when the full-length *b* variant of the PMCA3 pump was considered. As shown in Fig. 4c, d, overexpression of the wt PMCA3 variant *b* led to a statistically significant decrease in the formation of the Ca^2+^ microdomain under the PM compared to control cells, indicating a variant-dependent role of this PMCA isoform in shaping the sub-PM [Ca^2+^]. Interestingly, the E1081Q mutation completely abolished the observed phenotype of its wt counterpart (peak values ± SEM: 245,7 ± 7,77 μM, *n* = 31 for control cells; 187,1 ± 5,64 μM, *n* = 34 for the wt PMCA3b pump; 247,6 ± 8,77 μM, *n* = 33 for the E1081Q PMCA3b pump). These results could also be interpreted as a lack of difference in the sub-PM effect due the low expression level of the PMCA3b E1081Q mutant. However, the ability of this mutant to behave like the wt pump in counteracting the Ca^2+^ release from the intracellular stores or in regulating the total ER Ca^2+^ content independent on its expression level (Fig. 3d, e) prompted us to hypothesize that the specific ability to fine tune the [Ca^2+^] in the sub-PM domain could indeed be differentially affected. Taken collectively, these results depict the interesting variant-dependent picture in which the E1018Q mutation affected the Ca^2+^ extrusion ability of the PMCA3 pump by different means: in variant *a* through selectively increasing the Ca^2+^-clearance from the Ca^2+^ microdomains formed in the sub-PM region without affecting its ability to clear the Ca^2+^ deriving from the intracellular stores, while in variant *b* by selectively impinging capacity to reduce the [Ca^2+^] from the Ca^2+^ microdomains in the sub-PM domain. These results also highlight the potential different roles of the PMCA3 variants in the regulation of the Ca^2+^ beneath the PM, an aspect that deserves additional experiments.

### Allosteric Ca^2+^ binding on the PMCA C-terminal domain and effect of the E1081Q mutation

**Fig. 5:**
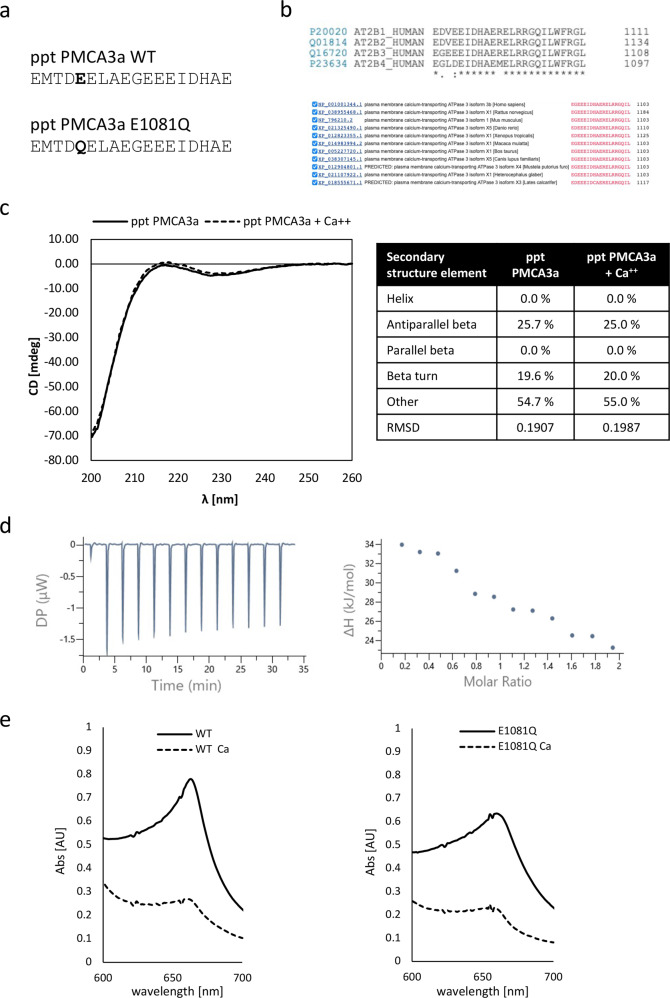
PMCA peptides-Ca^2+^ interaction studies. **a** Amino acidic sequences of wild-type PMCA3a 18mer (A18 [32]) and E1081Q mutant peptides, exploited for Ca^2+^ studies. **b** Sequence alignment showing the conservation of the residues around the mutated region among PMCA3 isoforms (1–4) and species. **c** Far UV CD spectra (left panel) of A18 peptide in absence (solid line) and presence (dashed line) of 1 mM free Ca^2+^, and their respective deconvolution (right panel) with the estimated secondary structure. **d** ITC raw (left panel) and blank-subtracted processed (right panel) data of peptide A18 - Ca^2+^ binding experiment. No fitting model was able to identify reasonable binding. **e** Absorbance spectra of Stains-all samples of A18 (left panel) and E1081Q mutant peptides (right panel) in the absence (solid line) and presence (dashed line) of 1 mM free Ca^2+^.

It had been previously shown that the C-terminal domain of the plasma membrane Ca^2+^ pump contain three high affinity Ca^2+^ binding sites [32] with possible regulatory roles. It was felt interesting to investigate whether the E1081Q mutation, specifically located within one of the regions of these potential allosteric Ca^2+^ binding sites, could somehow impact on the ability of this region to efficiently bind Ca^2+^ thus affecting a potential regulation of the pump. To this aim we designed two peptides corresponding to the A18 peptide synthesized in [32] (A18_wt) and the corresponding E1081Q mutated peptide (A18_E1081Q, Fig. 5a): this region is conserved across PMCA isoforms and across species (Fig. 5b). The binding of Ca^2+^ to synthetic wt and E1081Q peptides was thus explored by either the ITC assay and the specific probing with the Stains-all dye, a cationic carbocyanine dye commonly used as a valuable tool in the identification of potential Ca^2+^ binding proteins and as a sensitive method for probing Ca^2+^ binding [33–35]. As shown in Fig. 5c, the CD spectra of the wt peptide showed no difference in the presence and in the absence of Ca^2+^, suggesting that no major changes can be induced by Ca^2+^ in the secondary structure of the peptide. When the ITC was performed no binding was detected between the synthetic peptide and free Ca^2+^ within the concentrations range explored (48 μM peptide, 0–65 μM free Ca^2+^, Fig. 5d). On the other hand, indirect evidence of Ca^2+^ binding by the synthetic 18mer PMCA derived peptide was observed through a decrease of the absorption at the J-band after the addition of CaCl_2_ when using the Stains-all compound (Fig. 5e, left panel), similarly to what was previously reported by Hofmann and coworkers [32]. Furthermore, a comparable effect was observed when using the peptide bearing the mutation E1081Q (Fig. 5e, right panel), suggesting that the Ca^2+^ binding features of this PMCA peptide, if present in the tested conditions, are not significantly impacted by the above-mentioned mutation.

### Molecular modeling of the effect of the E1081Q mutation in PMCA3

**Fig. 6:**
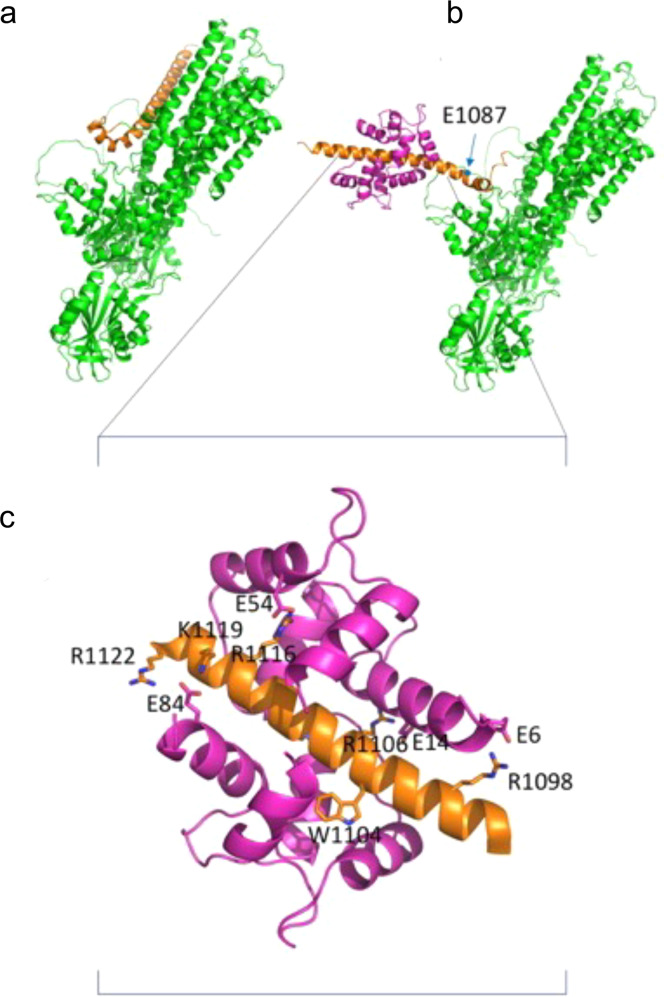
Hypothetical model of PMCA-calmodulin interaction. **a** Cartoon representation of the structure of human PMCA predicted by Alpha-fold [36]. The calmodulin-binding domain is colored orange. Only residues from 1074 to 1130, mostly a long a-helix, are present since the remaining C-terminal portion is predicted disordered. **b** The PMCA model with calmodulin bound (magenta). The calmodulin model was taken from the crystal structure of the Ca^2+^-bound calmodulin in complex with the myristoylated alanine-rich C kinase substrate (PDB ID 1IWQ, [48]) deprived from the bound peptide and the solvent. The orientation of the domain was obtained by rotating around the torsion angles of Glycine 1074, but its orientation in model B is completely hypothetical. **c** Detail of the area of interaction PMCA-calmodulin (residues from 1097 to 1122) and is characterized by the presence of several positively charged and hydrophobic residues, mostly neutralized by a corresponding negatively charged glutamate present on calmodulin. Tryptophan 1104, whose side chain is essential for the binding, is nicely placed in a hydrophobic cavity of the ligand.

To explore the effect of the E1081Q mutation on PMCA3 structure and function, homology modeling by using the SERCA pump as a template could not be used since, to date, it has proven impossible to model the C-terminal domain of the PMCA due to its flexible nature and to the disordered region at the C-terminal tail. We took advantage of the AlphaFold database [36], where the first structure prediction of the full-length PMCA3, including its CaM-BD, could be found. As shown in Fig. 6a, the structure of human PMCA predicted by Alpha-fold shows the CaM-BD in orange (residues from 1131 to the C-terminal end are predicted disordered and are not shown for clarity). Interestingly, the E1081 residue is located immediately upstream the CaM-BD. The PMCA model with calmodulin bound (magenta), obtained by submitting the model of the isolated calmodulin-binding domain along with the structure of calmodulin to the docking server Clus-Pro 2.0 [37], is shown in Fig. 6b. The structure of the complex emulates that of the starting crystal structure, with the calmodulin surrounding the PMCA helix with its two domains. Since the binding of calmodulin to the PMCA, cannot take place owing to strong steric hindrance, a different position of the CaM-BD was hypothesized. The region of interaction of the calmodulin-binding domain (Fig. 6c) includes more than 20 residues (from 1097 to 1122) and is characterized by the presence of several positively charged and hydrophobic residues, and by the absence of negatively charged residues. In our model, all the arginine residues are neutralized by a corresponding negatively charged glutamate present on calmodulin: R1098 → E6, R1106 → E14, R 116 → E54, R1122 (and K119) → E84. The only exception is represented by R1110, which is nevertheless close to Q1114. Tryptophan 1104, whose side chain is essential for the binding, is nicely placed in a hydrophobic cavity of the ligand. It is tempting to use the model to speculate that the E1081Q mutation could impact on the structure of the CaM-BD by affecting either its autoinhibition ability of the pump or that the binding of CaM would occur immediately downstream. The affinity of CaM, for example, might be enhanced, thus explaining the positive modulation of the pump activity.

## Discussion

Cerebellar ataxias are rare neurological disorders characterized by the lack of voluntary coordination of muscle and gait abnormality [38]. Alterations of cerebellar structures or a combination of cerebellar and extra-cerebellar lesions are the main cause and define the autosomal recessive, autosomal dominant or X-linked recessive [38] forms. Different mutations have been associated with inherited cerebellar ataxias, including those of the PMCA3 pump. As mentioned above, PMCA2 and PMCA3 are enriched in neurons [39], possibly due to their higher affinity and lower sensitivity for calmodulin [39, 40]. PMCA3 accumulates in the cerebellar region involved in motor control and sensory perception which is mainly constituted by Purkinje neurons [41]. As mentioned, a link between PMCA3 mutations and X-linked cerebellar ataxias has now been repeatedly described [22, 23, 26], the pump mutations being constantly accompanied by additional mutations in other proteins [22, 25]. In this study, we have described three novel mutations of the PMCA 3 pump and have further characterized the effects of one missense mutation (E1018Q) immediately upstream the CaM-BD of the neuron-enriched isoform 3 of the PMCA pump of two unrelated human patients suffering from neurodevelopmental (SCN1A) or neurodegenerative disorders (IBA57) and cerebellar dysfunction.

As mentioned in the Introduction, the PMCA pumps only offer a minor contribution to the global cytosolic Ca^2+^ homeostasis. Their main role is the regulation of Ca^2+^ signalling in selective sub-plasma membrane microdomains: the important point is that these microdomains host a number of important enzymes, which activity is directly or indirectly regulated by Ca^2+ 40^. The results described in this contribution underline once more the concept that the function of the PMCA pump, at least in specialized cells like neurons, is next to irrelevant to the global regulation of cytosolic Ca^2+^. It has been shown, for instance, that the ablation of PMCA4 isoform from cardiac cells has no effect on the Ca^2+^-dependent contractility properties of the cardiomyocytes [40]. It is, however, of paramount importance to the function of a number of Ca^2+^signaling events which are modulated by partners of the pump in the sub-plasma membrane microdomains [39]. The same situation could be for PMCA3 pump which by regulating the Ca^2+^ concentration in the microdomains, very likely, regulates the activity of resident enzymes, which could be so exquisitely sensitive to Ca^2+^ that subtle changes in Ca^2+^ concentration, also beyond the detection levels afforded by the present experimental protocols, would be sufficient to generate neuronal dysfunction [42, 43]. One more point could be added in this context. Ca^2+^ in cells must be regulated in an extremely precise way: no deviations from the optimal concentration levels -importantly, in either direction- can be tolerated [39, 44]. We have shown here that the E1081Q mutation affects the PMCA3 function in an isoform-dependent manner by decreasing and increasing the ability to fine tune the [Ca^2+^] in the sub-PM microdomains by the full-length *b* and the truncated *a* variant, respectively. Mechanistically this could be due to their different regulation of the activity by specific partners or by impaired allosteric regulation through differences in Ca^2+^ binding. Although we were able to replicate the Ca^2+^ binding to the A18 peptide by using the stains all, other A18-Ca^2+^ binding experiments failed to reveal any binding. Thus, whether this peptide binds Ca^2+^ or not was unclear, as was the impact of the E1081Q mutation on the Ca^2+^ binding feature of that region of the CTD of PMCA3. Possibly, however, the Ca^2+^ affinity of the A18 peptide could have been too low to reveal Ca^2+^ binding under the experimental conditions and/or, the length of peptide could have been insufficient to construct a Ca^2+^-binding module. It is felt that future work on these aspects of the defects of the PMCA3 pump that would include the development of currently unavailable experimental protocols, could possibly shed light on the link between PMCA pump defects and the ataxic phenotype.

## Materials and Methods

### Mutational analysis and site-direct mutagenesis

Targeted next-generation sequencing was performed by standard methods (Qiagen DNA extraction kit) to isolate genomic DNA from peripheral blood of the patient. Informed consent was obtained. Mutational analysis of genes associated with pediatric ataxia or candidate gene *ATP2B3* was performed as in [21]. The plasmid encoding the *a* and the *b* variants of mutated PMCA3 (R696H, E1081Q and R1133Q for the *a* variant, E1081Q for the *a* and *b* splice variants) were obtained by site-direct mutagenesis and cloned into pMM2 or pcDNA3 vector, respectively. Mutagenesis was performed using the QuickChange XL site-direct mutagenesis kit (Agilent) by using the following primers: h/rPMCA3 R696H ff 5’-cgaaaatgccagcatgctggcatcaca-3’; h/rPMCA3 R696H rev 5’-tgtgatgccagcatgctggcattttcg-3’; hPMCA3 E1081Q ff 5’-gacgagatgaccgaccaggagctggccgaa-3’; hPMCA3 E1081Q rev 5’-gccttcggccagctcctggtcggtcatctc-3’; rPMCA3 E1081Q ff 5’-gatgagatgactgatcaagagttggcggaa-3’; rPMCA3 E1081Q rev 5’-cccttccgccaactcttgatcagccatctc-3’; hPMCA3 R1133Q ff 5’-cagggtgctgtgcgccagcggtcttcggtc-3’; hPMCA3 R1133Q rev 5’-gaggaccgaagaccgctggcgcacagcacc-3’. The constructs were verified by DNA sequencing.

### Cell cultures and transfection

HeLa cells (ATCC) were grown in DMEM high glucose (Euroclone) with 10% Fetal bovine serum (GIBCO), 100U/ml penicillin (Euroclone) and 100 µg/ml streptomycin (Euroclone). Cells were seeded onto 13-mm glass coverslips (immunofluorescence and ER Ca^2+^) or 6-multiwell plates (western blot and cytosolic Ca^2+^) are allowed to grow to 70–80% confluence before transfection. Transfection was performed with the Ca^2+^ phosphate procedure (4 µg of total DNA for 13 mm coverslip and 12 µg/well for the 6-multiwell plate. For Ca^2+^ measurement cells were co-transfected with aequorin constructs targeted to different cell compartments (cytAEQ, erAEQ) and pcDNA3 empty vector or short/long PMCA3 wild type/mutated plasmids.

### Western blotting analysis

HeLa cells lysates were prepared 48 h after transfection and quantified by the Bradford assay (Bio-Rad), loaded on 8% SDS/PAGE Tris-HCl polyacrylamide gel and blotted onto Immobilon-P^SQ^ PVDF Membrane (Merck Millipore). The membrane was blocked for 1 h at room temperature using 5% non-fat dried milk (NFDM) in TBST (20 mM Tris-HCl, pH 7.4, 150 mM NaCl, 0.05% Tween-20) and incubated overnight with the primary antibody at 4 °C. Rabbit polyclonal anti-PMCA3 antibody, 1:1000 (PA1916, Thermo Fisher) and anti-β-actin, 1:30000 (A5441, Sigma) primary antibodies were used. Detection was performed with secondary HRP-conjugated anti-mouse or anti-rabbit IgG antibodies (sc-2005 and sc-2004, Santa Cruz Biotechnology) for 1 hr at room temperature followed by incubation with the chemiluminescent reagent Luminata HRP substrate (Merck Millipore).

### Immunocytochemistry analysis

Forty-eight hours after transfection, HeLa cells were processed for immunofluorescence. The cells were washed twice with phosphate-buffered saline (PBS: 140 mM NaCl, 2 mM KCl, 1.5 mM KH_2_PO_4_, 8 mM Na_2_HPO_4_ pH 7.4) and fixed for 20 minutes with 3,7% formaldehyde in PBS. Cells permeabilization was performed by 20 minutes incubation with 0,1% Triton X-100 in PBS, followed by 30 minutes wash with 1% gelatin (type IV, from calf skin, Sigma) in PBS. The coverslips were then incubated for 60 minutes at 37 °C in a wet chamber with a rabbit polyclonal anti-PMCA3 antibody (PA1916, Thermo Fisher) at a 1:100 in PBS. Staining was revealed by the incubation with specific AlexaFluor 488-labeled anti-mouse secondary antibody, 1:50 (A-11001, Life technologies) for 45 minutes at room temperature. Fluorescence was detected with a Leica SP5 confocal microscope and analysed by ImageJ software.

### Aequorin Ca^2+^ measurements

Cytosolic, ER and sub-PM Ca^2+^ measurements were carried out on a PerkinElmer EnVision plate reader as previously reported [25].

### Peptide synthesis

Wt and E1081Q mutant peptides corresponding to the 18-residues upstream of PMCA3 CaM-BD were synthesized as previously described [27]. The sequences of the wt and the mutated peptides, which carried a E or a Q residue at position 1081 were the following: EMTDEELAEGEEEIDHAE and EMTDQELAEGEEEIDHAE.

### Yeast manipulation, strains and plasmids

Yeast cultures and genetic manipulations have been performed according to standard methods [45]. Yeast strain K616 (Mat α; *pmr1::HIS3*; *pmc1::TRP1*; *cnb1::LEU2*; *ade2-1*; *ura3-1*) [46] have been used after transformation and selection with pYES2-derivative plasmids, carrying either wild-type or mutant PMCA3b, which allow the overexpression of the pump in galactose-based medium. Both PMCA3-pYES2 plasmids, carrying the wild-type and the PMCA3-ΔC_ter_ mutant, have been previously described [22]. Mutagenesis has been performed (Stratagene, Cedar Creek, TX) by using the following primers E1081Q-for (5’-gatgagatgactgatcaagagttggcggaa-3’) and E1081Q-rev (5’-cccttccgccaactcttgatcagccatctc-3’). Plasmids have been verified by sequencing. The expression of PMCA3 protein has been verified by Western blotting, as previously described [22], and total protein content has been obtained by TCA-based solubilization of yeast cells [47]. Immunoblots have been performed by using the rabbit polyclonal anti-PMCA3 antibody, 1:1000 (PA1916, Thermo Fisher).

### Functional complementation assay in K616 yeast cells

Yeast K616 cells expressing either wild-type, E1081Q or ΔCter mutant PMCA3 pumps have been used as described previously [22, 24]. Exponentially growing yeast cells have been serially diluted and spotted on selective medium containing glucose (PMCA3 repressed) supplemented with 10 mM CaCl_2_, or Galactose (PMCA3 induced) supplemented with 10 mM EGTA, reflecting permissive or selective conditions for yeast K616 viability, respectively. Yeast K616 cells carrying the empty plasmid have been considered as negative experimental control, whereas those expressing the PMCA3-ΔCter mutant were used as positive control. Plates were then incubated at the standard temperature (30 °C) for 3–6 days. Functional assays have been performed by testing 3–5 independent clones from the yeast transformation plate, and further confirmed by 3 independent transformations of yeast cells (n > 10).

### Structural modeling

The structure of human PMCA is predicted by Alpha-fold [36]. Only residues from 1074 to 1130 are present since residues from 1131 to the C-terminal end are predicted disordered and are not shown. The calmodulin-PMCA complex was obtained by submitting the model of the isolated calmodulin-binding domain along with the structure of calmodulin to the docking server Clus-Pro 2.0 [37]. The calmodulin model was taken from PDB ID 1IWQ [48] deprived from the bound peptide and the solvent. The prediction with calmodulin bound to the long helix of the PMCA domain in the area defined by the experimental studies using isolated peptides [49] was considered as the correct one. Due to strong steric hindrance with the other helices of PMCA, binding of CaM cannot take place with the a-helix in the original position, therefore a different conformation of PMCA with the calmodulin-binding domain in a different position was hypothesized.

### Secondary structure analysis

Circular Dichroism measurements were performed with a Jasco spectropolarimeter. Far-UV CD spectra were collected using cells of 0.1 cm path-length. Data were acquired at a scan speed of 20 nm min^−1^ and at least three scans were averaged. Peptides were used at a concentration of 0.5 mg ml^−1^, in a 5 mM Ammonium bicarbonate, pH 7, DMSO 0.5%. Measurements in the presence of Ca^2+^ were performed adding 1 mM CaCl_2_, final concentration. Experiments were performed at room temperature. The secondary structure content of A18_wt peptide in the absence and presence of free Ca^2+^ was calculated using the online CD spectrum deconvolution software Bestsel (https://bestsel.elte.hu/index.php) [50].

### Isothermal Titration Calorimetry

ITC measurements were carried out at 25 °C on a MicroCal PEAQ-ITC Instrument. The titrant and sample solutions were made from the same stock ITC buffer solution (2 mM MOPS pH 7.2, 30%, v/v, ethylene glycol), All the used solutions were preventively filtered through Chelex resin (Bio-Rad) to remove free Ca^2+^, and thoroughly degassed before each titration. The solution in the cell contained A18_wt peptide resuspended in ITC buffer at a concentration of 48 μM and was stirred at 750 rpm to ensure rapid mixing. The titration experiment started with a first 0.4 μL injection of titrant, followed by 13 injections of 13 μL of the same (500 μM CaCl_2_ in ITC buffer), with a spacing time between each injection of 150 seconds to allow complete equilibration. A background titration, consisting of identical titrant solution and buffer solution in the sample cell in absence of peptide, was subtracted to account for heat of dilution. The data were analyzed with the MicroCal PEAQ-ITC Analysis Software. No fitting model was able to identify a binding.

### Stains-all measurements

Stains-all spectra acquisitions were carried out as previously described [32]. Briefly, Stains-all was dissolved in ethylene glycol at a concentration of 500 μM, while the spectra acquisition solution was 2 mM MOPS pH 7.2, 30% ethylene glycol (spectra buffer). The peptides, in the presence or absence of CaCl_2_, were incubated at 4 °C for 1 h with rotation, with 20 μM Stains-all in spectra buffer, at a Stains-all to peptide molar ratio of 10:1. The spectra were recorded immediately after the incubation, measuring their absorbance from 700 to 400 nm using a spectrophotometer.

### Statistical analysis

All the data are representative of at least three independent experiments unless otherwise indicated. Values are expressed as mean ± SEM. Statistical significance was determined using the multiparametric one-way ANOVA test. A *p* value ≤0.05 was considered statistically significant.

## Supplementary information

Figure S1

Figure S2

aj-checklist

## Data Availability

The experimental data sets generated during the current study are available from the corresponding author upon reasonable request. No applicable resources were generated during the current study.
