# Supplementary figures and images for: The ataxia-linked E1081Q mutation affects the sub-plasma membrane Ca2+-microdomains by tuning PMCA3 activity
Source: Cell Death Dis. 2022 Oct 7;13(10):855. doi: 10.1038/s41419-022-05300-y (PMC9546857; doi:10.1038/s41419-022-05300-y)

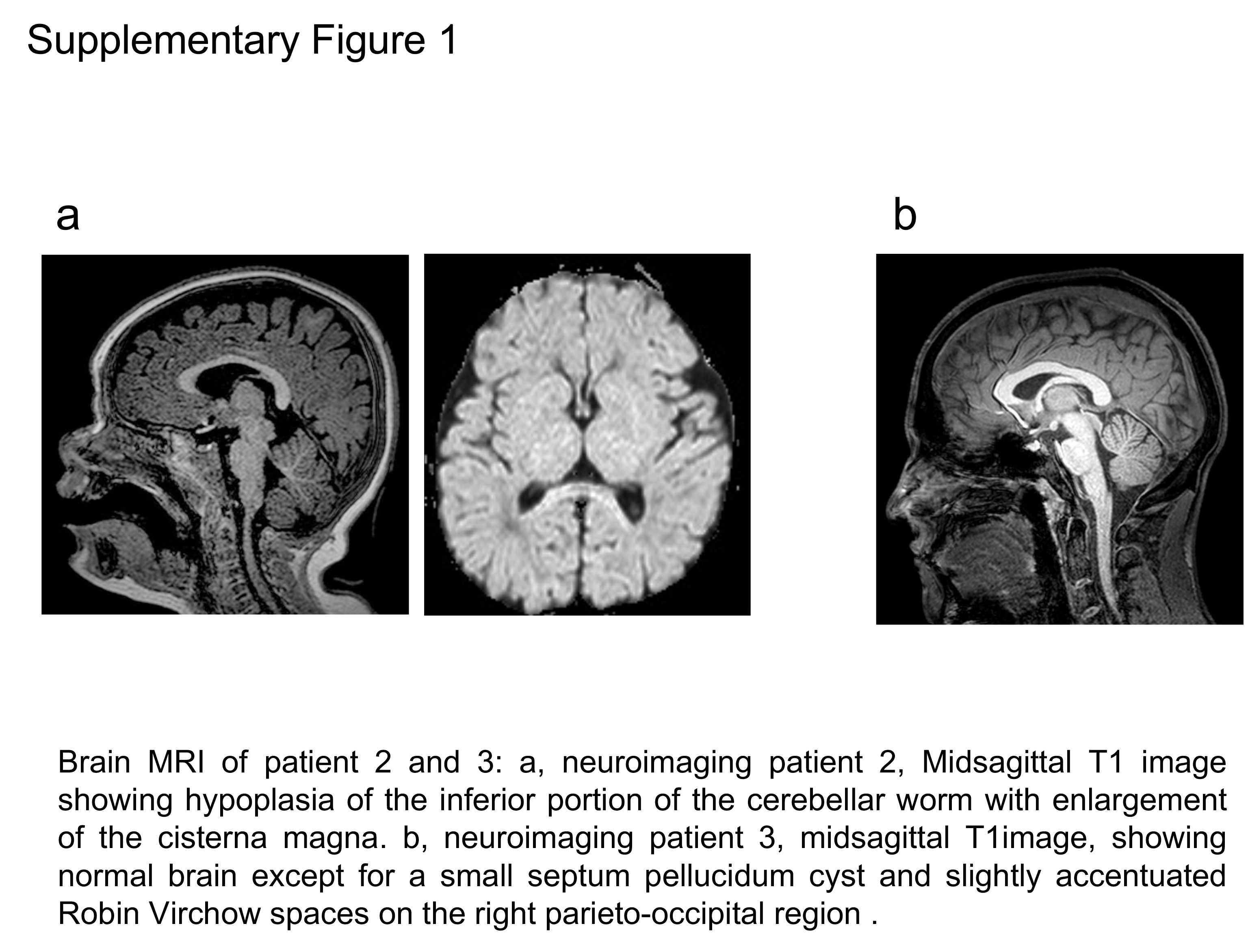

Supplement: Supplementary file 1 — Figure S1 [file 41419_2022_5300_MOESM1_ESM.tif]

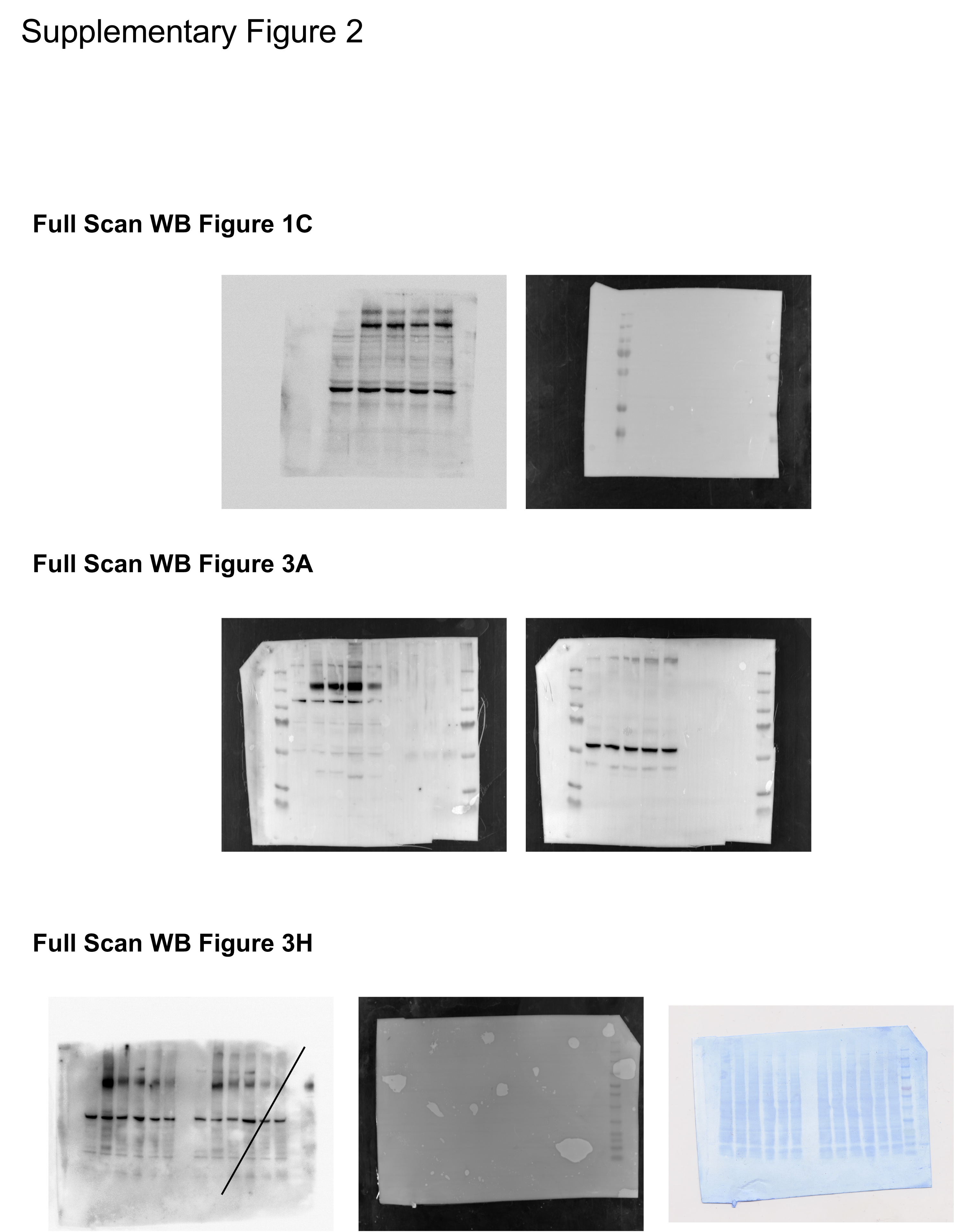

Supplement: Supplementary file 2 — Figure S2 [file 41419_2022_5300_MOESM2_ESM.tif]
